# ThHSFA1 Confers Salt Stress Tolerance through Modulation of Reactive Oxygen Species Scavenging by Directly Regulating *ThWRKY4*

**DOI:** 10.3390/ijms22095048

**Published:** 2021-05-10

**Authors:** Ting-Ting Sun, Chao Wang, Rui Liu, Yu Zhang, Yu-Cheng Wang, Liu-Qiang Wang

**Affiliations:** 1 State Key Laboratory of Tree Genetics and Breeding, Key Laboratory of Tree Breeding and Cultivation of the State Forestry Administration, Research Institute of Forestry, Chinese Academy of Forestry, Beijing 100091, China; sun1226236202@126.com (T.-T.S.); liuruizjj0715@163.com (R.L.); zhangyu7009@126.com (Y.Z.); 2 Beijing Academy of Forestry and Pomology Sciences, Beijing Engineering Research Center for Deciduous Fruit Trees, Key Laboratory of Biology and Genetic Improvement of Horticultural Crops (North China), Ministry of Agriculture and Rural Affairs, Beijing 100093, China; 3 State Key Laboratory of Tree Genetics and Breeding, Northeast Forestry University, Harbin 150040, China; wzyrgm@163.com (C.W.); wangyucheng@ms.xjb.ac.cn (Y.-C.W.); 4 Co-Innovation Center for Sustainable Forestry in Southern China, Nanjing Forestry University, Nanjing 210037, China

**Keywords:** antioxidant enzyme, heat shock element, heat shock transcription factor, ROS, salt stress, *Tamarix* *hispida*, *ThHSFA1*

## Abstract

Heat shock transcription factors (HSFs) play critical roles in several types of environmental stresses. However, the detailed regulatory mechanisms in response to salt stress are still largely unknown. In this study, we examined the salt-induced transcriptional responses of *ThHSFA1*-*ThWRKY4* in *Tamarix* *hispida* and their functions and regulatory mechanisms in salt tolerance. ThHSFA1 protein acts as an upstream regulator that can directly activate *ThWRKY4* expression by binding to the heat shock element (HSE) of the *ThWRKY4* promoter using yeast one-hybrid (Y1H), chromatin immunoprecipitation (ChIP), and dual-luciferase reporter assays. *ThHSFA1* and *ThWRKY4* expression was significantly induced by salt stress and abscisic acid (ABA) treatment in the roots and leaves of *T*. *hispida*. ThHSFA1 is a nuclear-localized protein with transactivation activity at the *C*-terminus. Compared to nontransgenic plants, transgenic plants overexpressing *ThHSFA1* displayed enhanced salt tolerance and exhibited reduced reactive oxygen species (ROS) levels and increased antioxidant enzyme activity levels under salt stress. Therefore, we further concluded that *ThHSFA1* mediated the regulation of *ThWRKY4* in response to salt stress in *T*. *hispida*.

## 1. Introduction

Salinity and secondary soil salinization have become serious ecological environmental problems worldwide, affecting plant growth and production by causing osmotic imbalance, mineral deficiency, and overall toxicity [1]. To maintain adequate growth under salinity stress conditions, plants alter their physiological, biochemical, molecular, and cellular processes through activating and integrating the expression of many genes and tend to reestablish ionic and reactive oxygen species (ROS) homeostasis [2,3]. Therefore, transcriptional regulation plays crucial roles in mediating signaling networks to allow plants to cope with various stress conditions, and transcription factors (TFs), such as HSF (heat shock transcription factor), WRKY, and NAC (NAM, ATAF1/2 and CUC2) family members, are known to be involved [4,5,6,7,8].

Plant HSFs are commonly involved in responses to several types of environmental stresses, such as heat, salt, drought, and oxidation [3,9,10,11,12,13]. The *HSF* gene family is large and consists of many members, including 25 members in Arabidopsis, 52 in soybean, 56 in wheat and 47 in poplar, according to the plant transcription factor database (PlantTFDB). Similar to many other TFs, HSFs contain a conserved *N*-terminal DNA-binding domain (DBD), which can bind to cis-acting heat shock element (HSE) recognition sequences in their target gene promoters [14]. The hydrophobic heptad repeat pattern of hydrophobic amino acid residues (HR-A/B) is essential for oligomerization [15]. Based on their domains, HSFs can be subdivided into three classes (A, B, and C). Class A members, HSFAs, possess a *C*-terminal activation domain characterized by short peptide motifs (AHAs) and function as transcriptional activators, whereas class B and C HSFs lack a defined activation domain. HSFBs may act as transcriptional repressors, and the function of HSFCs function remains elusive [16,17,18,19]. In response to abiotic stress, most *HSF*s are regulated by the heat stress response, especially *HSFA1*, *HSFA2*, and *HSFA6*, in tomato, Arabidopsis, and wheat [20,21,22]. Furthermore, overexpression of *AtHSFA1b* in Arabidopsis increases water productivity and enhances resistance to drought stress conditions by directly regulating *HSE1b*-containing genes [23]. *AtHSFA4A* was identified as a regulatory factor conferring salt and oxidative stress tolerance in Arabidopsis [24]. *CmHSFA4*, a homolog of *AtHSFA4a*, confers salt stress tolerance in chrysanthemum by maintaining cellular Na^+^/K^+^ ion and ROS homeostasis [3]. The salt and drought tolerance of *AtHsfA6a*-overexpressing plants is enhanced via the abscisic acid (ABA) signaling pathway, and these plants exhibit increased expression of downstream stress-responsive genes [10]. *AtHSFA6b* participates in ABA-mediated salt and drought resistance and the heat response and acts as a positive regulator [21]. In addition to the roles of these HSFs in herbaceous plants, *Populus euphratica PeHSF* overexpression in tobacco maintains leaf ROS homeostasis and enhances antioxidant enzyme activity levels under salt stress conditions [4]. *PuHSFA4a* serves as a positive regulator and promotes zinc tolerance through coordinated regulation of root development and the antioxidant system by directly activating the expression of *PuGSTU17* and *PuPLA* [25].

The WRKY TF family is one of the largest families of plant-specific proteins and plays important roles in the response to different abiotic stresses and the ABA signaling pathway. For example, overexpression of wheat *TaWRKY2* and *TaWRKY19*, soybean *GmWRKY12*, *GmWRKY16* and *GmWRKY54*, or sweet potato *IbWRKY2* in transgenic plants results in enhanced salt and drought tolerance [26,27,28,29,30]. *OsWRKY45* alleles play different roles in ABA signaling, *OsWRKY45-1* is insensitive to salt stress in rice, and *OsWRKY45-2* negatively regulates the response to salt stress [31]. Transgenic plants overexpressing cotton *GhWRKY6*-like gene or sweet potato *IbWRKY2* display enhanced salt tolerance by regulating the ABA signaling pathway and ROS scavenging [30,32]. Recently, the expression of *FtWRKY46* from buckwheat, *DgWRKY4* and *DgWRKY5* from chrysanthemum, and *GarWRKY5* from cotton in transgenic plants was shown to confer enhanced tolerance to salt stress by modulating ROS clearance, antioxidant enzyme activity, and stress-related gene expression [6,33,34,35]. Although many studies have reported that *WRKY* gene*s* are involved in different types of abiotic stress by enhancing downstream stress-responsive gene expression, it is not clear which TFs can regulate *WRKY*s in response to salt stress.

*Tamarix hispida*, a species of the genus Tamarix, is highly tolerant to salinity and drought and can be used as an excellent material for studying tree physiology and identifying stress tolerance genes. Many well-documented studies on stress-related genes have proved the physiological and molecular mechanisms of salt tolerance in *T*. *hispida*, such as *WRKY*, *bZIP* (basic leucine zipper protein), *bHLH* (basic helix–loop–helix), *NAC*, *MYB*, and *PIP* (plasma membrane intrinsic protein) [7,36,37,38,39,40]. Our previous studies have proved that ThWRKY4, as a dimeric protein, can form homodimers and heterodimers with ThWRKY2 and ThWRKY3 and enhance salt stress tolerance in transgenic Arabidopsis by modulating ROS scavenging and the expression of stress-responsive genes [7,41]. We further analyzed the promoter sequences of *ThWRKY4* and found a cis-acting HSE in the promoter. Therefore, we speculated whether HSF binds to *ThWRKY4* promoter regions containing cis-acting HSE and directly activates *ThWRKY4* expression. In this study, we identified that *ThHSFA1* directly regulates *ThWRKY4*. Additionally, *ThHSFA1* confers salt tolerance by regulating ROS scavenging and the ABA signaling pathway. The data showed that *ThHSFA1* and *ThWRKY4* are involved in the same physiological and molecular regulatory pathways of the salt stress response.

## 2. Results

### 2.1. Identification of ThHSFA1 as an Upstream Regulator of ThWRKY4

Our previous study demonstrated that *ThWRKY4* can be regulated by ABF (ABRE binding factor) and Dof (DNA binding with one finger) and other WRKYs [7]. Further analysis of the *ThWRKY4* promoter sequence revealed that it contains a cis-acting HSE using the PLACE database, so we speculated that *ThWRKY4* might be regulated by HSFs. To identify the TFs, the yeast one-hybrid (Y1H) assay was used to screen the cDNA library of *T. hispida*. After the putative positive clones were re-streaked on high-stringency SD/−Leu/−Trp/−His (TDO) selective medium supplemented with 40 mM 3-AT (3-amino-1,2,4-triazole) to validate growth. The cDNA inserts from pGADT7 vectors were sequenced and analyzed via BLAST programs. We found that the positive yeast clone harboring an *HSF* gene grew better than the other yeast clones. The *HSF* gene consists of a 1545 bp open reading frame (ORF) encoding a 518-amino-acid protein, which contains several homeobox domains, including a conserved DBD, an intermediate HR-A/B, a putative nuclear localization signal (NLS) region, and an AHA motif (Figure S1A). Sequence alignment and phylogenetic analysis showed that the HSF is similar to other known HSFAs and shared the highest amino acid sequence identity (~49%) with AtHSFA1D of *Arabidopsis*; thus, it was named ThHSFA1 (Figure S1B).

**Figure 1:**
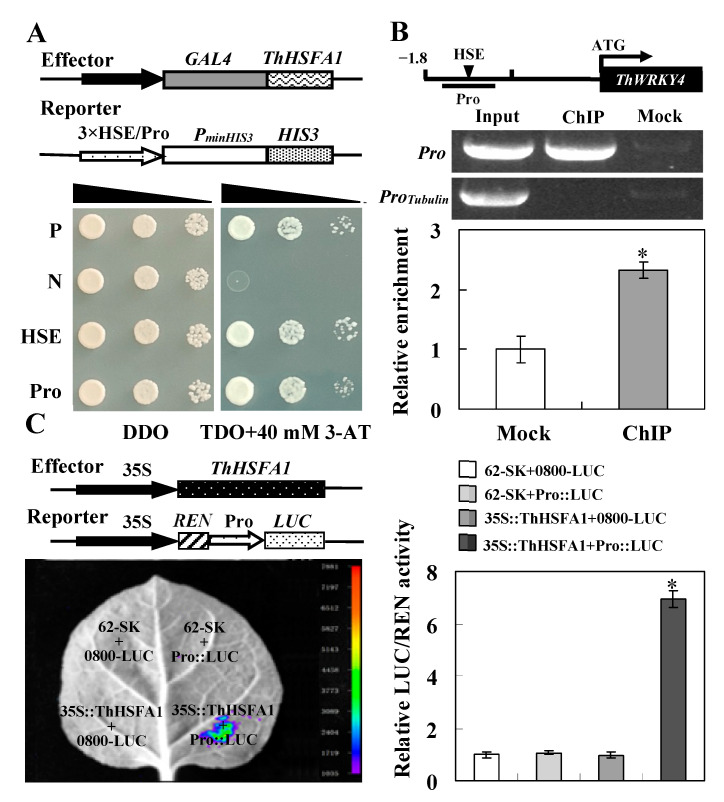
Identification of the upstream regulator ThHSFA1 of ThWRKY4. (**A**) Y1H assay. Three tandem copies of cis-acting HSE and the *ThWRKY4* promoter fragment (Pro) were inserted into the pHIS2 vector. The reporter and effector constructs were co-transformed into yeast Y187 cells, and transformed cells were identifying by spotting serial dilutions of yeast onto selective medium SD/−Leu/−Trp (DDO) and SD/−Leu/−Trp/−His (TDO) containing 3-amino-1,2,4-triazole (3-AT). P: positive control (p53HIS2 + pGAD-53); N: negative control (p53HIS2 + pGAD-ThHSFA1). (**B**) ChIP assay. Simplified *ThWRKY4* gene structure showing the ThHSFA1 binding regions (Pro) and the cis-acting HSE. qPCR analysis of the abundance of the *ThWRKY4* promoter fragment. Input, chromatin preparation before immunoprecipitation; ChIP, immunoprecipitated with an anti-GFP antibody; Mock, immunoprecipitated without an anti-GFP antibody. (**C**) Dual-luciferase reporter assay. A schematic of the recombinant effector (35S::ThHSFA1) and reporter (Pro::LUC) constructs used for the transient expression assays is shown. The effector and reporter vectors were simultaneously co-transformed into *N*. *benthamiana* leaves, and the luminescence intensity was measured. Tobacco leaves injected with 0800-LUC+ 62-SK, Pro::LUC+62-SK and 0800-LUC+35S::ThHSFA1 were used as negative controls. Luciferase activity is expressed relative to the control levels (value set at 1.0). The error bars represent ± SD from multiple biological replicates, and the asterisks indicate significant differences (*, *p* < 0.05).

To confirm that the ThHSFA1 protein binds to the cis-acting HSE sequence in the *ThWRKY4* promoter and regulates the expression of *ThWRKY4*, the promoter fragment of *ThWRKY4* (Pro) containing the cis-acting HSE was amplified and inserted into the pHIS2 vector, and the full-length cDNA of *ThHSFA1* was cloned into the pGADT7-Rec2 vector. The positive transformants harboring *ThHASF1* and the *ThWRKY4* promoter fragment grew well on TDO medium containing 3-AT (Figure 1A). In addition, the truncated *ThWRKY4* promoter was detected when chromatin was prepared before immunoprecipitation (Input) and 35S::ThHSFA1-GFP was precipitated (ChIP^+^), whereas the almost no promoter fragment was detected when chromatin was immunoprecipitated without an anti-GFP antibody (Mock). The promoter fragment was enriched approximately 2.5-fold in the immunoprecipitated sample compared with the mock sample (Figure 1B). Moreover, tobacco leaves co-transformed with vector controls (62-SK+0800-LUC) and negative controls (62-SK+Pro::LUC and 35S::ThHSFA1+0800-LUC) exhibited invisible luciferase luminescence. Luciferase luminescence was observed when 35S::ThHSFA1 and Pro::LUC were co-transformed into tobacco leaves, and the relative LUC/REN activity was approximately 7 folds that of the leaves subjected to transformation with control vectors (Figure 1C). Taken together, these results confirm that ThHSFA1 protein binds to the cis-acting HSE in the *ThWRKY4* promoter to regulate *ThWRKY4* expression in *T*. *hispida*.

### 2.2. ThHSFA1 and ThWRKY4 Show Similar Expression Patterns under Abiotic Stress

**Figure 2:**
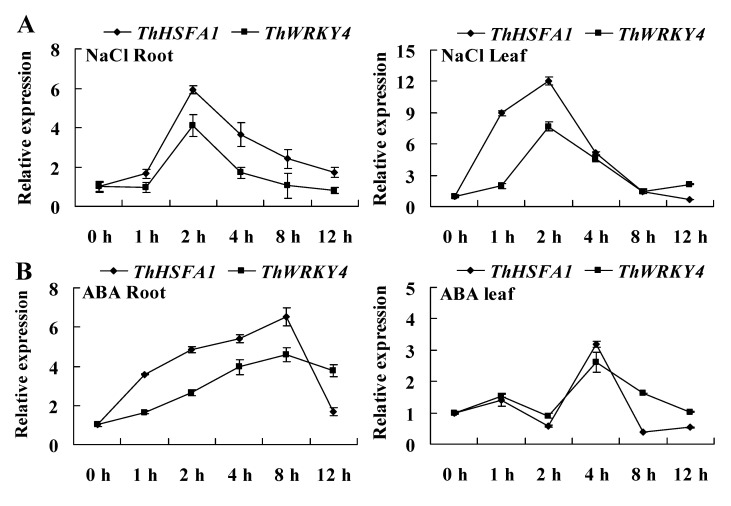
Expression patterns of *ThHSFA1* and *ThWRKY4* in response to abiotic stress. The transcript levels of *ThHSFA1* and *ThWRKY4* in roots and leaves from *T*. *hispida* seedlings treated with 300 mM NaCl (**A**) or 120 µM ABA (**B**) for the indicated times were measured by RT-qPCR. A fresh water only-treated control was established in parallel. The reference gene *Thtubulin* was used as an internal control. The error bars represent ± SD, which were calculated from multiple biological replicates.

The expression of *ThHSFA1* was induced by salt stress and ABA treatment, although these patterns differed between the roots and leaves. *ThHSFA1* expression in leaves and roots rapidly increased after the onset of salt exposure, reaching a maximum level at 2 h, and then was decreased at the subsequent time points (Figure 2A). Under ABA treatment, the expression of *ThHSFA1* in roots steadily increased in the early period of stress and gradually increased to the peak expression level at 8 h of stress, although a decline was observed at 12 h. In leaves, the *ThHSFA1* transcript level was slightly increased or decreased after 2 h of ABA treatment, markedly increased to the highest level at 4 h, and was then significantly decreased at 8 and 12 h (Figure 2B). Next, we investigated whether salt and ABA stress also induced *ThWRKY4* expression in the roots and leaves of *T. hispida*. Reverse transcription quantitative PCR (RT-qPCR) results showed that the expression of *ThHSFA1* was not only induced by salt stress and ABA treatment but also similar to the expression pattern of *ThHSFA1* under abiotic stress. These results further suggest that ThHSFA1 protein regulates the expression of *ThWRKY4* under abiotic stress and indicate that *ThHSFA1* and *ThWRKY4* may share a regulatory mechanism.

### 2.3. ThHSFA1 Is Localized in the Nucleus and Has Transcriptional Activation Activity

**Figure 3:**
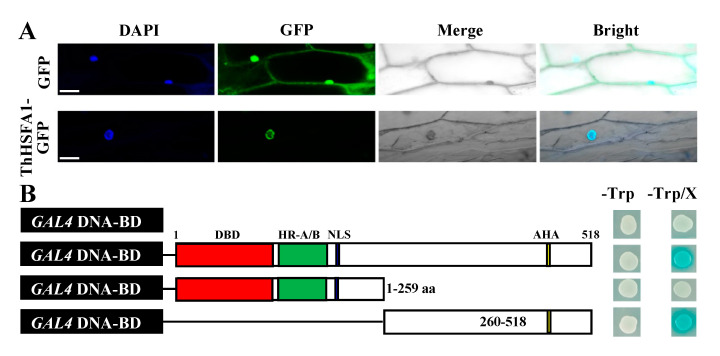
Subcellular localization and transcriptional activation assay of ThHSFA1. (**A**) Subcellular localization of ThHSFA1 protein. The 35S::GFP (control) and 35S::ThHSFA1-GFP were transformed into onion epidermal cells and visualized by DAPI staining. Scale bars = 10 µm. (**B**) Transcriptional activation activity of ThHSFA1 protein. The full-length and fragment cDNA of *ThHSFA1* were fused to the GAL4 DNA-binding domain in the yeast vector pGBKT7 and expressed in Y2HGold yeast cells. The transformed yeast cells were plated on SD/−Trp and SD/−Trp/X-α-Gal media and then subjected to a β-gal assay.

To confirm the subcellular localization of ThHSFA1 protein, the 35S::ThHSFA1-GFP fusion protein and 35S::GFP (control) were expressed in onion epidermal cells. GFP fluorescence was mainly detected in the nuclei of cells transformed with 35S::ThHSFA1-GFP. In contrast, GFP fluorescence of the control vector was distributed throughout the cells, including the nucleus and cytoplasm (Figure 3A). These results indicate that ThHSFA1 is a nuclear protein.

To assess the transactivation activity of ThHSFA1, the full-length and partial cDNA of *ThHSFA1* were cloned into the pGBKT7 vector and introduced into Y2HGold yeast cells (Figure 3B). On SD/−Trp medium with or without X-α-Gal, all positive transformants containing the recombinant or control vectors grew well. The transformants containing the full-length and *C*-terminal domains of ThHSFA1 appeared blue, whereas the transformants containing pGBKT7 or the *N*-terminal domain did not appear on the medium. These findings revealed that ThHSFA1 functions as a transcriptional activator and that the *C*-terminal polypeptide containing the AHA motif has transcriptional activation ability.

### 2.4. ThHSFA1 Positively Regulates Salt Stress Tolerance

**Figure 4:**
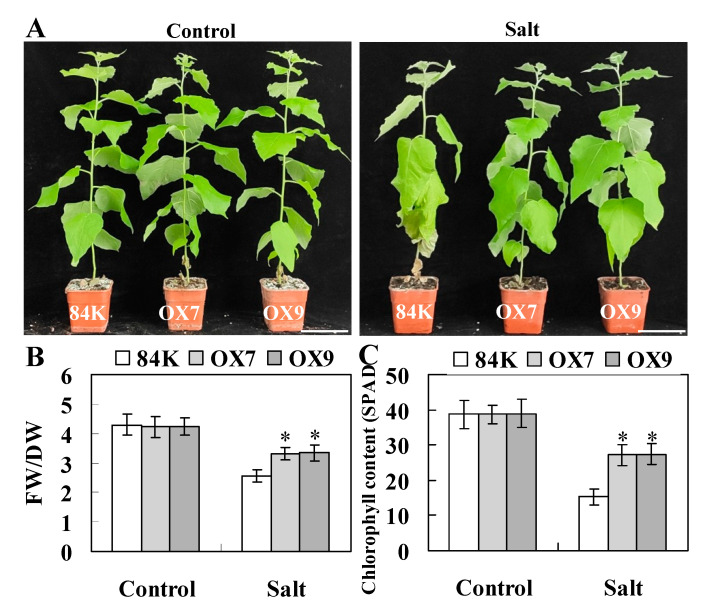
*ThHSFA1* enhances salt tolerance in transgenic poplar. The phenotype (**A**), fresh weight/dry weight (FW/DW) ratio (**B**), and chlorophyll content (**C**) of nontransgenic 84K and transgenic (OX) plants. Four-week-old nontransgenic 84K and transgenic plants cultivated in vitro were grown in soil for 30 days and subsequently irrigated with 0 (control) or 200 mM NaCl (salt) every 2 days for 10 days. The error bars represent ± SD from multiple biological replicates, and the asterisks indicate significant differences between nontransgenic 84K and transgenic plants (*, *p* < 0.05). Scale bars = 10 cm.

To investigate the potential biological functions of *ThHSFA1*, we generated 11 transgenic poplar lines overexpressing *ThHSFA1* and confirmed the altered gene expression by RT-qPCR (Figure S2). Two independent overexpression lines (OX7 and OX9) with high transcript levels of *ThHSFA1* were selected for further analysis, along with the corresponding nontransgenic 84K poplar. To assess salt tolerance, poplars of each genotype were subjected to salt stress (200 mM NaCl) for 10 days. Under control growth conditions, the transgenic and nontransgenic 84K plants were morphologically indistinguishable, and the plant height, basal diameter, fresh weight/dry weight (FW/DW) ratio, and chlorophyll content of each poplar genotype were not significantly different (Figure 4A and Figure S3). After 10 days of salt stress, the nontransgenic 84K plants displayed more serious salt damage than the OX lines, and most of the leaves of the nontransgenic 84K plants were seriously wilted, whereas those of the transgenic lines still appeared normal (Figure 4A). However, the plant height and basal diameter of the poplars of each genotype were not significantly different (Figure S3). Furthermore, the FW/DW ratio and chlorophyll content were generally reduced in all the genotypes under salt stress, but these reductions were less marked in the OX lines than that of nontransgenic 84K plants (Figure 4B,C). These observations suggested that overexpression of *ThHSFA1* enhances the salt tolerance of transgenic poplars.

### 2.5. ThHSFA1 Reduces ROS Accumulation and Enhances Antioxidant Enzyme Activity

To further investigate the functions of *ThHSFA1*, we also generated three kinds of transient transgenic Tamarix plants, including transgenic Tamarix overexpressing (OE), RNAi-silenced (RNAi) *ThHSFA1* plants, and transformed with an empty pROKII vector (VC). The advantage of the transient transformation method is that it has no positional effect and that the expression levels of transiently expressed genes in plants are similar throughout the transformant [42]. However, it was not possible to build different transgenic lines as stable transformants. The expression level of *ThHSFA1* was obviously higher in OE plants and significantly lower in RNAi plants then in VC plants, and the difference in expression levels was more obvious under salt stress and ABA treatment (Figure S4), indicating that these genetically transformed *T*. *hispida* plants are suitable for further study.

**Figure 5:**
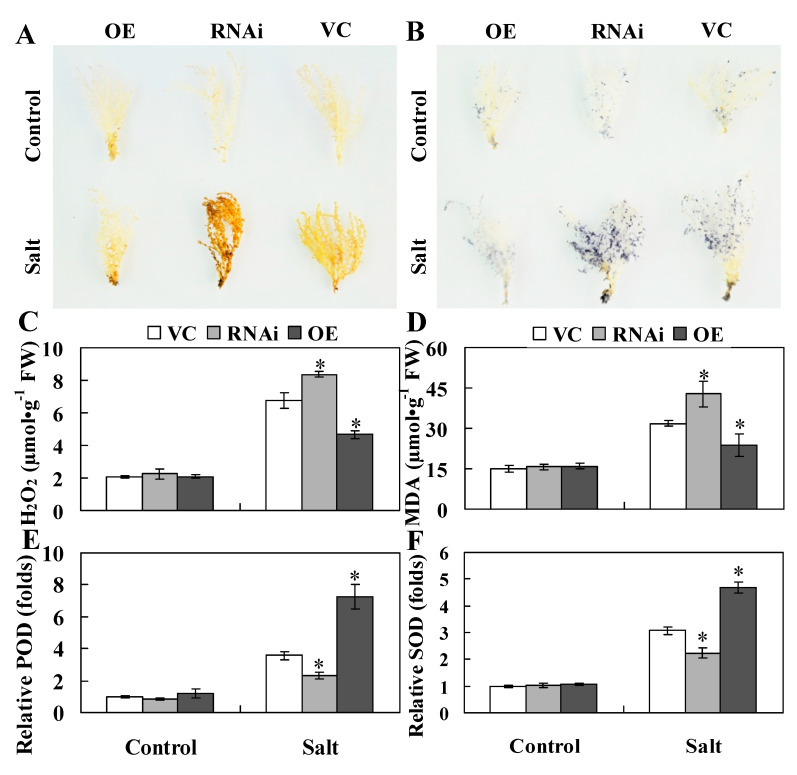
*ThHSFA1* decreases ROS levels in *T. hispida* plants under salt stress. (**A**,**B**) DAB and NBT staining were used to measure H_2_O_2_ and O_2_^•−^ levels. (**C**–**F**) H_2_O_2_ and O_2_^•−^ content and relative POD and SOD activity levels in *T. hispida* plants. The transiently transformed *T*. *hispida* plants were grown on half-strength Murashige–Skoog (1/2 MS) medium supplemented with 0 (control) or 150 mM NaCl (salt) for 24 h, and leaves were collected for histochemical staining and physiological analyses. VC: empty pROKII-transformed *T. hispida* plants; RNAi: *T. hispida* plants with transient RNAi-mediated silencing of *ThHSFA1*; OE: *T. hispida* plants with transient overexpression of *ThHSFA1*. The error bars represent ± SD from multiple biological replicates, and the asterisks indicate significant differences between the VC and RNAi or OE plants (*, *p* < 0.05).

**Figure 6:**
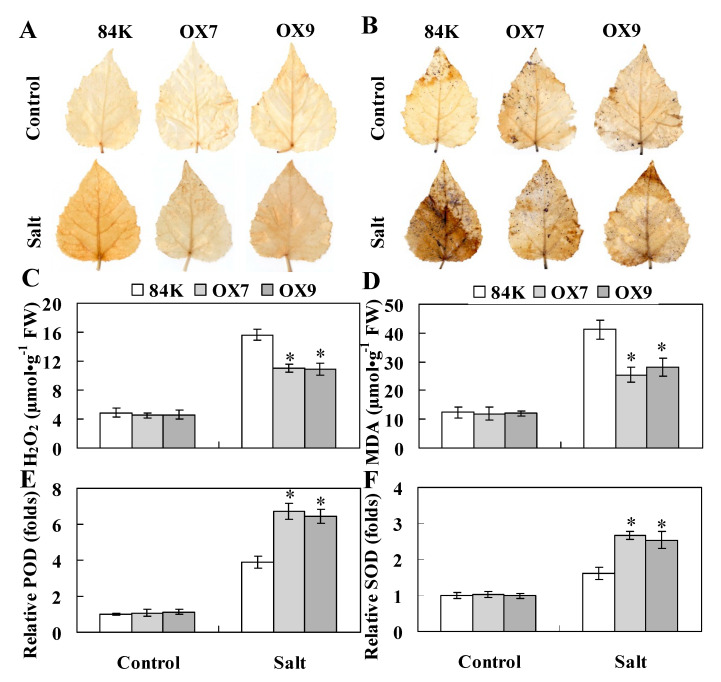
*ThHSFA1* enhances ROS scavenging capability of transgenic poplar plants under salt stress. Leaves were collected from nontransgenic 84K and transgenic poplars before and after salt stress and used for histochemical staining including DAB (**A**) and NBT (**B**) staining, and physiological analyses, including measurements of H_2_O_2_ (**C**) and MDA (**D**) content and relative POD (**E**) and SOD (**F**) activity levels. Poplar plants were cultivated and subjected to salt stress (200 mM NaCl) every 2 days for 10 days as described in Figure 4. The error bars represent ± SD from multiple biological replicates, and the asterisks indicate significant differences between nontransgenic 84K and transgenic plants (*, *p* < 0.05).

A previous study reported that constitutive expression of *ThWRKY4* in Arabidopsis confers enhanced salt tolerance by improving ROS scavenging capability and antioxidant enzyme activity [7]. ThHSFA1 protein is an upstream regulator of *ThWRKY4*, so we speculated that ThHSFA1 may be involved in the same physiological regulatory pathway of the salt stress response. We first monitored the cellular levels of hydrogen peroxide (H_2_O_2_) and superoxide (O_2_^•−^) in different types of transiently transformed *T. hispida* plants and genotyped poplars by histochemical staining with 3,3′-diaminobenzidine (DAB) and nitroblue tetrazolium (NBT), with the intensity of staining representing the amount of H_2_O_2_ and O_2_^•−^. Under control growth conditions, there was no obvious difference between the transgenic and the corresponding control Tamarix (VC) and poplar (84K) plants. After salt stress, the leaf staining of transgenic Tamarix (OE) and poplar (OX) plants was weaker than those of the corresponding control plants (Figure 5A,B and Figure 6A,B). Furthermore, the blue and brown spots of transgenic Tamarix plants with knockdown of *ThHSFA1* (RNAi) were darker than those of VC plants (Figure 5A,B). Quantitative measurement of H_2_O_2_ content revealed similar results and further demonstrated that H_2_O_2_ content in the transgenic lines of Tamarix and poplar was lower than that in the corresponding control plants (Figure 5C and Figure 6C). In addition, malondialdehyde (MDA) is a valid indicator of cytomembrane oxidative damage [43]. Under control growth conditions, MDA content was similar in transgenic and nontransgenic plants. However, transgenic Tamarix (OE) and poplar (OX) plants overexpressing *ThHSFA1* displayed lower MDA levels than the corresponding control plants under salt stress conditions (Figure 5D and Figure 6D). Both histochemical staining and quantitative measurement indicated that overexpression of *ThHSFA1* in transgenic plants reduced ROS accumulation under salt stress conditions.

Antioxidant enzymes, such as peroxidase (POD) and superoxide dismutase (SOD), are major ROS scavengers and play crucial roles in ROS homeostasis [44]. To further illustrate the roles of *ThHSFA1* in ROS detoxification, we measured the activities of POD and SOD. Under control growth conditions, POD and SOD activity levels in transgenic Tamarix and poplar plants were similar to those in nontransgenic plants. However, under salt stress, *ThHSFA1*-overexpressing Tamarix plants had distinctly higher POD and SOD activity levels than transgenic plants transformed with the control vector (VC), and transgenic Tamarix plants with knockdown of *ThHSFA1* (RNAi) showed significantly lower POD and SOD activity levels than VC plants (Figure 5E,F). Likewise, POD and SOD activity levels were similar between transgenic and nontransgenic 84K plants under control growth conditions, the activity levels of these two enzymes in OX7 and OX9 lines were significantly higher than those in nontransgenic 84K plants under salt stress (Figure 6E,F). These results indicated that *ThHSFA1* overexpression enhanced antioxidant enzyme activity in transgenic plants under salt stress.

## 3. Discussion

### 3.1. ThHSFA1 Binds the ThWRKY4 Promoter and Activates the Expression of ThWRKY4

WRKY genes have been widely demonstrated to be involved in plant responses to abiotic stresses, such as drought, salt, and cold [45]. Most of them function as transcriptional activators and enhance stress tolerance by regulating stress-responsive gene expression. For example, Tartary buckwheat *FtWRKY46*, chrysanthemum *DgWRKY4* and *DgWRKY5*, and cotton *GarWRKY5* improve tolerance to salt stress [6,33,34,35]. Similarly, our previous study reported that *ThWRKY4* enhances salt tolerance through the regulation of downstream target genes [7]. However, whether *WRKY* genes are regulated by other TFs requires further investigation. In the present study, we found a specific cis-acting HSE in the promoter sequence of *ThWRKY4* and identified a class A HSFs, ThHSFA1, that can bind to the HSE (Figure 1A). ThHSFA1 protein contains an *N*-terminal DBD responsible for HSE recognition, an HR-A/B involved in oligomerization, an AHA for transcription factor activity, and a putative NLS region (Figure S1). Our data further confirmed that ThHSFA1 is a nuclear protein and has transcriptional activation activity (Figure 2). It acts as an upstream regulator that directly binds to the HSE of the *ThWRKY4* promoter, thus activating the expression of *ThWRKY4* (Figure 1). In addition, the expression patterns of *ThHSFA1* and *ThWRKY4* in the roots and leaves of Tamarix under salt stress and ABA treatment are quite similar (Figure 2), further supporting the molecular regulation mode. Consistent with our results, the expression of both *PeHSF* and *PeWRKY1* is both induced by salt stress, and PeHSF protein can activate *PeWRKY1* expression by binding to the cis-acting HSE of the *PeWRKY1* promoter [5].

### 3.2. ThHSFA1 Enhances Tolerance to Salt Stress

Previous studies have shown that different HSF family members are involved in various stresses by regulating the expression of stress-related target genes, including high temperature, drought, oxidative stress, and pathogen infection [46]. A few class A HSFs have been reported to play critical roles in the salt stress response [3]. *AtHSFA2* and *AtHsfA7b*-overexpressing Arabidopsis plants display enhanced salt stress tolerance [8,12]; additionally, in Arabidopsis, *AtHSFA4A* regulates salt and oxidative stress responses [24], and *AtHsfA6a* increases salt and drought stress tolerance [10]. Transgenic overexpression of *OsHsfA2e* and *TaHsfA6f* in Arabidopsis results in tolerance to high salt stress [47,48]. In addition, *PeHSF*-overexpressing tobacco exhibits significantly improved salt tolerance [4]. In this study, *ThHSFA1* expression was notably induced by salt and ABA stresses in roots and leaves, and transgenic Tamarix and poplar plants overexpressing *ThHSFA1* showed enhanced tolerance to salt stress. Under salt stress conditions, ectopic expression of *ThHSFA1* in poplars was less serious, and plants subjected to salt stress had a higher FW/DW ratio and chlorophyll content than nontransgenic 84K plants (Figure 4). These results suggested that *ThHSFA1* has positive regulatory roles in the salt stress response.

### 3.3. ThHSFA1 Confers Salinity Tolerance by Regulating ROS Homeostasis

Our previous study proved that heterologous expression of *ThWRKY4* in transgenic Arabidopsis leads to enhanced tolerance to salt and ABA stresses by regulating ROS scavenging and the expression of stress-responsive genes [7]. In the present study, ThHSFA1 protein directly regulates *ThWRKY4* expression in *T*. *hispida*, the expression of both *ThHSFA1* and *ThWRKY4* was induced by salt stress and ABA treatment, and these two genes showed similar expression patterns (Figure 2), implying that *ThHSFA1* and *ThWRKY4* confer salt tolerance by the same mode of action. *ThHSFA1* overexpression in transgenic plants decreased ROS accumulation under salt stress. Moreover, transgenic plants exhibited higher antioxidant enzyme (POD and SOD) activity than the corresponding control plants (Figure 5 and Figure 6), indicating that ThHSFA1 plays a pivotal role in the regulation of ROS homeostasis and antioxidant defenses under salt stress. Previous studies have confirmed the relationship between HSFs and ROS scavenging enzymes in the response to salt stress. For example, *PeHSF* improves the salt tolerance of transgenic plants with higher activity levels of antioxidant enzymes, including ascorbate peroxidase (APX), glutathione reductase (GR), and glutathione peroxidase (GPX) [4]. *CmHSFA4*-overexpressing plants displayed elevated activity levels of antioxidant enzymes under salt stress, such as SOD, APX, and catalase (CAT) [3]. It was recently demonstrated that *AtHSFA7b* positively mediates salt stress tolerance by regulating the expression of stress-related genes encoding antioxidant enzymes (SOD, POD, and GST) [8].

## 4. Materials and Methods

### 4.1. Plant Materials and Stress Treatments

*Tamarix hispida* was used for gene cloning, transgenic analysis, and expression analysis. Tamarix tissue was grown on Murashige–Skoog (MS) (PhytoTechnology Laboratories, Kansas, KS, USA) solid medium (pH 5.8–6.0) that consisted of 25 g L^−1^ sucrose and 7 g L^−1^ agar in a phytotron (24 °C, 14 h light/10 h dark photoperiod). Four-week-old seedlings were transplanted into pots filled with turf peat and sand (2:1 *v*/*v*) in a growth chamber (24 °C, 14 h light/10 h dark photoperiod, 70–75% relative humidity). Uniformly developed 2-month-old seedlings were treated with 300 mM NaCl or 120 µM ABA for 0, 1, 2, 4, 8, or 12 h. A fresh-water-only-treated control was established in parallel. After these treatments, the roots and leaves were harvested and stored at –80 °C for gene expression level analysis.

Hybrid poplar (*Populus alba* × *P*. *glandulosa* cv. ‘84K’) was used for genetic transformation and functional analysis. 84K poplar tissues were grown on 1/2 MS solid medium (pH 5.8–6.0, 30 g L^−1^ sucrose, 5 g L^−1^ agar, 0.05 mg L^−1^ indolebutyric acid, and 0.02 mg L^−1^ naphthylacetic acid) in a phytotron (25 °C, 16 h light/8 h dark photoperiod, 50 µM m^−2^ s^−1^ light intensity).

*Nicotiana benthamiana* was used for luciferase reporter assays. Seeds of *N*. *benthamiana* were surface sterilized in 5% (*v*/*v*) sodium hypochlorite and germinated on 1/2 MS solid medium containing 0.8% agar. One-week-old seedlings were transplanted into pots filled with soil and perlite (3:1) in a growth chamber at 22 °C with a 16 h light/8 h dark photoperiod and 70–75% relative humidity.

### 4.2. RNA Extraction and RT-qPCR Analysis

Total RNA was extracted from the collected materials using the CTAB (hexadecyltrimethylammonium bromide) method. First-strand cDNA was synthesized using the PrimeScript^TM^ RT Reagent Kit (TaKaRa, Dalian, China), and RT-qPCR was conducted on a Roche Light Cycler 480 (Roche Applied Science, Penzberg, Germany) with a SYBR Premix Ex Taq^TM^ Kit (TaKaRa, Dalian, China) according to the manufacturer’s instructions. Each sample was analyzed in triplicate, and four technical repeats were used for each sample. The primers used for RT-qPCR are listed in Table S1.

### 4.3. Y1H Assay

A cis-acting HSE was found in the promoter of *ThWRKY4*, which functions as a transcription factor to positively regulate the salt stress response [7], suggesting that *ThWRKY4* may be regulated by HSFs. To identify the upstream regulator of *ThWRKY4*, three tandem copies of HSE were cloned into the reporter vector pHIS2 as a bait to screen a *T*. *hispida* cDNA library using the Matchmaker™ Yeast One-Hybrid Library Construction and Screening Kit (Clontech, Palo Alto, CA, USA) following the manufacturer’s instructions. To investigate the interaction between the cis-acting HSE and candidate regulator, the promoter fragment of *ThWRKY4* (260 bp) containing the HSE (named Pro) was inserted into the pHIS2 vector, and the interaction was evaluated using the Y1H assay. The Y1H assay was repeated three times, and representative results are shown. The primers used for the Y1H assay are shown in Table S1.

### 4.4. ChIP Assay

Based on Y1H analysis, ThHSFA1 protein is a putative upstream regulator that activates the expression of *ThWRKY4*. To further confirm this result, the full-length cDNA of *ThHSFA1* excluding the termination codon was fused to the green fluorescent protein (GFP) reporter gene driven by the CaMV 35S promoter to generate vector 35S::ThHSFA1-GFP. The recombinant vector was introduced into *Agrobacterium tumefaciens* strain GV3101, which was transiently transformed into 6-week-old *T*. *hispida* seedlings as described previously [39,49]. In brief, whole seedlings were soaked in a solution (1/2 MS medium+5% (*w*/*v*) sucrose+150 μM acetosyringone+0.6 OD_600_ *A. tumefaciens*+0.01% (*w*/*v*) Tween-20, pH 5.8) and shaken at 130 rpm for 6 h at 25 °C. Then, the seedlings were transferred to 1/2 MS solid medium and grown for 48 h. Transgenic *T*. *hispida* was used for ChIP, and ChIP was conducted as described previously [50,51]. The sonicated chromatin was immunoprecipitated with an anti-GFP antibody (ChIP) or immunoprecipitated without an anti-GFP antibody (Mock). ChIP-qPCR was used to study the fold enrichment of the studied promoter fragments and performed with the following program: 95 °C for 30 s, followed by 45 cycles of 94 °C for 5 s, 60 °C for 30 s, dissociation at 95 °C for 5 s, and then 60 °C for 60 s. The PCR products were detected via 1.2% agarose gel electrophoresis. The *ThTubulin* promoter fragment was used as an internal control. Three independent biological replicates were performed for the ChIP assay. The details of the primers used for the ChIP assay are shown in Table S1.

### 4.5. Dual-Luciferase Reporter Assay

The full-length cDNA of *ThHSFA1* was cloned into the effector vector pGreenII 62-SK driven by the CaMV 35S promoter to generate 35S::ThHSFA1, and the *ThWRKY4* promoter fragment (260 bp) used in the Y1H assay was cloned into the reporter vector pGreenII 0800-LUC to generate Pro::LUC. The recombinant vectors and control plasmids were introduced into *A. tumefaciens* strain GV3101, and the effector and reporter vectors were co-transformed into the fully developed leaves of 6-week-old *N*. *benthamiana* seedlings according to the method described by Yao et al. [52]. D-Luciferin (10 µM) was sprayed onto the tobacco leaves and then photographed using an LB985 NightSHADE fluorescence imaging system (Berthold Technologies, Bad Wildbad, Germany). Relative dual-luciferase (LUC/REN) activity was determined using a GloMax 20/20 luminometer (Promega, WIsconsin, WI, USA) and a Dual-Luciferase Assay Kit (Promega, Wisconsin, USA) following the instruction manual. The experiments were performed at least three times with six technical repeats. The primer sequences used for the dual-luciferase reporter assay are shown in Table S1.

### 4.6. Bioinformatics Analysis of ThHSFA1

HSF proteins from different plant species were searched against the NCBI database. The amino acid sequence of ThHSFA1 protein and its homologs were aligned with ClustalW using BioEdit software and adjusted manually. A phylogenetic tree was constructed with MEGA 5.05 using the neighbor-joining method, and the internal branch support was estimated with 1000 bootstrap replicates.

### 4.7. Subcellular Localization and Transcriptional Activation Analysis of ThHSFA1

To determine the subcellular localization of ThHSFA1 protein, the constructed vector 35S::ThHSFA1-GFP and control vector 35S::GFP were separately transformed into live onion epidermal cells using the biolistic bombardment method. After transformed and incubated on MS medium for 24 h at 22 °C in the dark, the transformed cells were visualized using confocal laser scanning microscopy (LSM700, Zeiss, Jena, Germany). To stain onion epidermal cell nuclei, 4′,6-diamidino-2-phenylindole (DAPI, 10 μg mL^−1^) in phosphate-buffered saline was used.

For transcription activity analysis of ThHSFA1 protein, the full-length and partial cDNA of *ThHSFA1* (encoding the *N*-terminus and *C*-terminus) were cloned into the yeast expression vector pGBKT7 and fused the GAL4 DBD (Clontech, Palo Alto, CA, USA). All constructs and the pGBKT7 vector (negative control) were introduced into Y2HGold yeast cells, which were cultured on SD/−Trp and SD/−Trp/X-a-Gal media for 2–3 days at 30 °C and then subjected to the β-galactosidase assay. The experiments were performed three times. All primers used are shown in Table S1.

### 4.8. Generation of Transgenic Plants

The full-length cDNA of *ThHSFA1* was cloned into the plant expression vector pMDC32 driven by the CaMV 35S promoter and introduced into *A*. *tumefaciens* strain GV3101. The transformation of 84K poplar was performed as described previously [53]. Putative transgenic plants were identified on selective medium supplemented with hygromycin (3 mg L^−1^) and verified by RT-qPCR with the *PagActin* gene.

The full-length cDNA of *ThHSFA1* was cloned into the plant expression vector pROKII driven by the CaMV 35S promoter to generate the vector 35S::ThHSFA1. The sense and antisense sequences of partial cDNA were cloned into the RNAi vector pFGC5941 to generate RNAi::ThHSFA1. The recombinant and control (pROKII) vectors were introduced into *A*. *tumefaciens* strain GV3101, which was transiently transformed into 6-week-old *T*. *hispida* seedlings [39,49]. *ThHSFA1* expression in the leaves of different transformed *T*. *hispida* plants was examined by RT-qPCR. All primers used are listed in Table S1.

### 4.9. Salt Stress Tolerance Assay

*ThHSFA1*-transformed and nontransgenic 84K poplar plants were propagated via in vitro microcutting. Shoot segments of transgenic and nontransgenic 84K poplars (~3 cm in length with 2–3 young leaves) were cut from sterilized seedlings and cultivated on 1/2 MS solid medium. To observe the growth of poplar plants under salt stress, 4-week-old transgenic and nontransgenic 84K plants were transplanted into plastic pots with a mixture of soil and perlite, grown for 30 days in a greenhouse at 25 °C, and irrigated subsequently with 0 (control) or 200 mM NaCl (salt treatment) solution every 2 days for 10 days. These plants were photographed, and the plant height, basal diameter, and fresh and dry weights were measured. Chlorophyll content in the 6^th^–8^th^ leaves of each genotype was measured using a portable chlorophyll analyzer (SPAD-502 Plus, Konica Minolta, Japan). The treatment experiments were repeated three times, and at least 20 plants of each genotype were used per experiment.

### 4.10. Histochemical Staining and Physiological Trait Measurements

The transiently transformed *T. hispida* seedlings were grown on 1/2 MS solid medium supplemented with 150 mM NaCl for 24 h. Poplar plants were cultivated and subjected to salt treatment every 2 days for 10 days as mentioned above. The young branches of *T*. *hispida* and the 6^th^–8^th^ leaves from the tip of poplars were collected and used for histochemical staining and physiological analysis. The experiments were performed three times, and at least 20 plants were used for each experiment.

The production of H_2_O_2_ and O_2_^•−^ was measured in situ using DAB and NBT staining according to the method described by Ding et al. [54]. In brief, approximately 20 young branches of *T*. *hispida* and leaves of poplar were incubated with DAB (0.1 mg mL^−^^1^) or NBT (0.1 mg mL^−^^1^) solution in the dark at 28 °C. After staining for 8 h, the stained leaves were incubated in a solution of 75–95% ethanol to remove chlorophyll and then photographed. The H_2_O_2_ and MDA content, and POD and SOD activity levels were measured using specific kits (Solarbio Science and Technology Co., Ltd., Beijing, China) following the manufacturer’s instructions.

## 5. Conclusions

In summary, this work revealed an important molecular regulatory mechanism involving *ThHSFA1-ThWRKY4* mediated salt stress tolerance in *T*. *hispida*. We further demonstrated that overexpression of *ThHSFA1* in Tamarix and poplar confers salt tolerance by modulating ROS scavenging capability and antioxidant enzyme activity. Further investigations are required to clarify whether *ThHSFA1* regulates other downstream stress-related genes and mediates ROS homeostasis in *T*. *hispida* under salt stress.

## Data Availability

*Tamarix hispid*a, Hybrid poplar (*Populus alba* × *P. glandulosa* cv. ‘84K’), and *Nicotiana benthamiana* plants were used in this study, and were kept in our laboratory (State Key Laboratory of Tree Genetics and Breeding, Key Laboratory of Tree Breeding and Cultivation of the State Forestry Administration, Research Institute of Forestry, Chinese Academy of Forestry, Beijing 100091, China).

## Supplementary Materials

The following are available online at https://www.mdpi.com/article/10.3390/ijms22095048/s1. Figure S1: Sequence alignment and phylogenetic analyses of ThHSFA1 and other plant HSF proteins. (A) Alignment of the amino acid sequences of some HSF proteins. Identity within the same amino acid is indicated by shading. The homeobox domains include the DBD, HR-A/B, NLS region, and AHA. (B) A phylogenetic tree of HSFA proteins was constructed by MEGA 5.05 using the neighbor-joining method. The numbers beside each node represent bootstrap values based on 1000 replications, Figure S2: Expression analysis of *ThHSFA1* in transgenic poplar by RT-qPCR. RT-qPCR results showing *ThHSFA1* expression in 11 transgenic overexpression poplar lines (OX). Parallel analysis of the *PagActin* gene was carried out for normalization to the amount of added template. The error bars represent ± SD, which were calculated from multiple biological replicates, Figure S3: Effects on the growth of transgenic poplar overexpressing *ThHSFA1*. Plant height and basal diameter of nontransgenic 84K and *ThHSFA1*-transformed poplars under control and salt stress conditions. Poplar plants were cultivated and subjected to salt treatment every 2 days for 10 days as described in Figure 4. The error bars represent ± SD from multiple biological replicates, Figure S4: RT-qPCR analysis of the expression of *ThHSFA1* in different transgenic *T. hispida* plants. The expression of *ThHSFA1* was determined under normal control conditions and treatment with 150 mM NaCl or 40 μM ABA for 24 h. The expression level of *ThHSFA1* in VC plants under control conditions was used as the calibrator (designed as 1). VC: the empty pROKII transformed *T. hispida* plants; RNAi: transiently RNAi-silencing *ThHSFA1* in *T. hispida* plants. OE: transiently overexpressing of *ThHSFA1* in *T. hispida* plants. The error bars represent ± SD, which were calculated from multiple biological replicates, Table S1: Primer sequences used in this study.
