# Supplementary material for: ThHSFA1 Confers Salt Stress Tolerance through Modulation of Reactive Oxygen Species Scavenging by Directly Regulating ThWRKY4
Source: Int J Mol Sci. 2021 May 10;22(9):5048. doi: 10.3390/ijms22095048 (PMC8126225; doi:10.3390/ijms22095048)
Supplement: Supplementary file 1 [file ijms-22-05048-s001.zip › ijms-1192954-supplementary.pdf]

International Journal of *Molecular Sciences*

MDPI

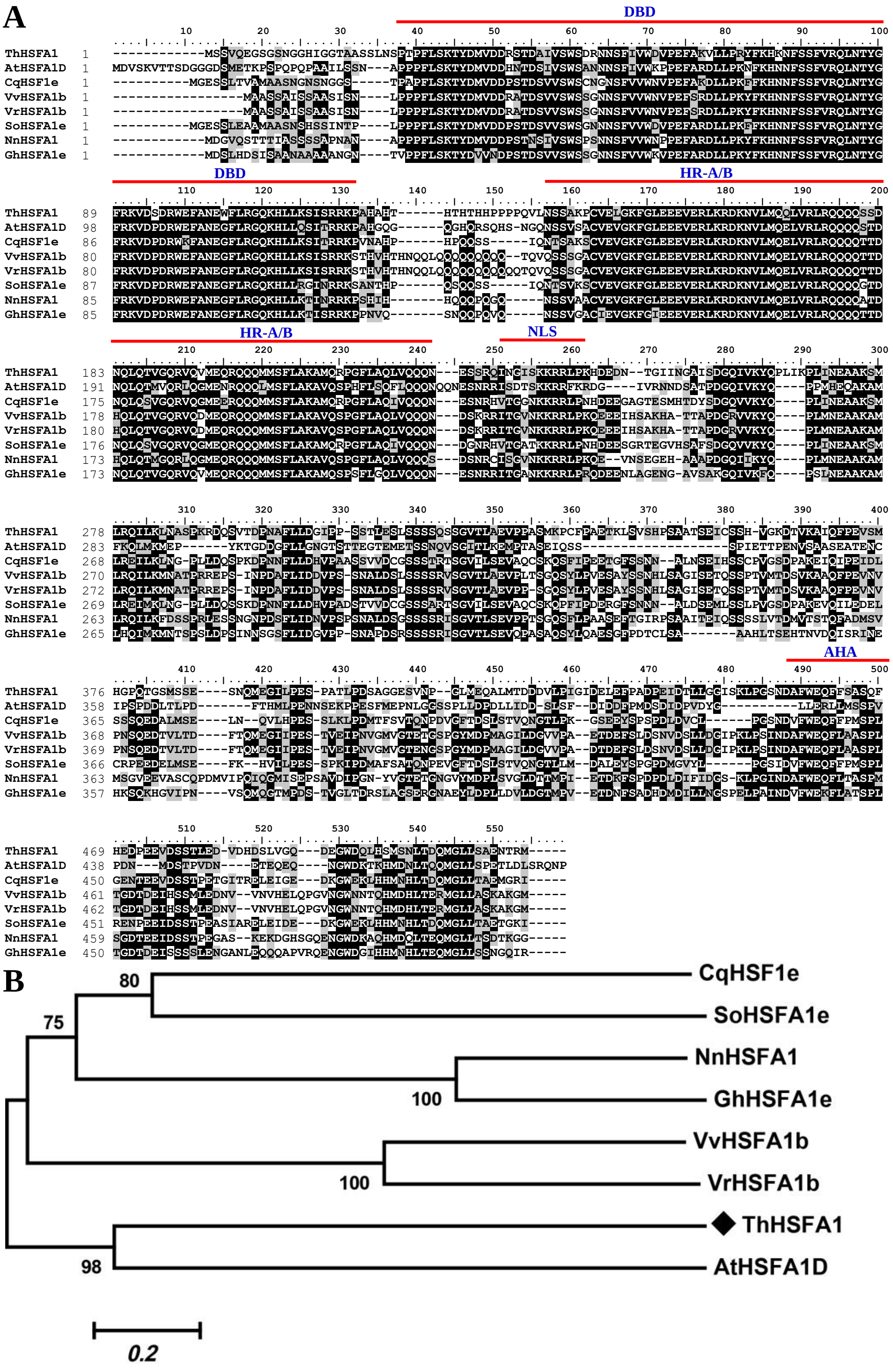

Figure S1. Sequence alignment and phylogenetic analyses of ThHSFA1 and other plant HSF proteins. (**A**) Alignment of the amino acid sequences of some HSF proteins. Identity within the same amino acid is indicated by shading. The homeobox domains include the DBD, HR-A/B, NLS region, and AHA. (**B**) A phylogenetic tree of HSFA proteins was constructed by MEGA 5.05 using the neighbor-joining method. The numbers beside each node represent bootstrap values based on 1000 replications.

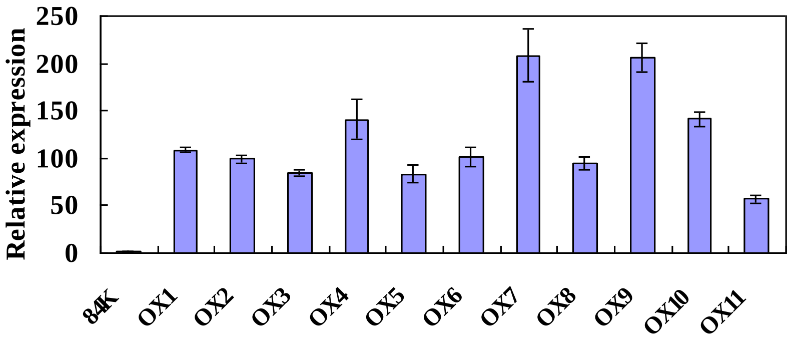

Figure S2. Expression analysis of *ThHSFA1* in transgenic poplar by RT-qPCR. RT-qPCR results showing *ThHSFA1* expression in 11 transgenic overexpression poplar lines (OX). Parallel analysis of the *PagActin* gene was carried out for normalization to the amount of added template. The error bars represent ± SD, which were calculated from multiple biological replicates.

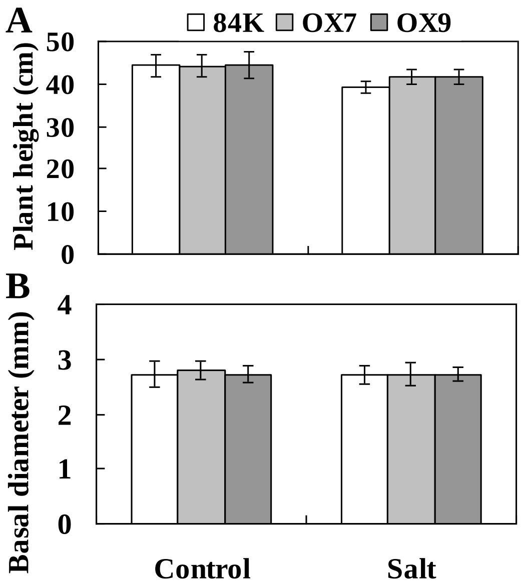

Figure S3. Effects on the growth of transgenic poplar overexpressing *ThHSFA1*. Plant height and basal diameter of nontransgenic 84K and *ThHSFA1*-transformed poplars under control and salt stress conditions. Poplar plants were cultivated and subjected to salt treatment every 2 days for 10 days as described in Figure 4. The error bars represent ± SD from multiple biological replicates.

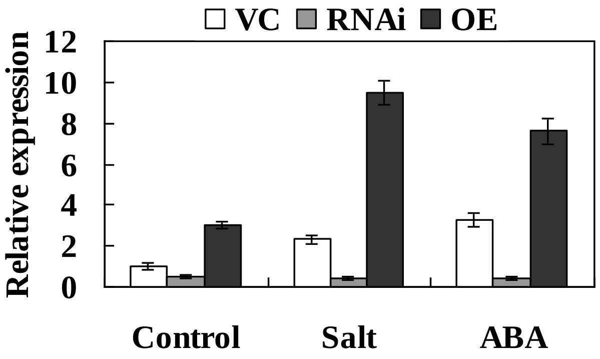

Figure S4. RT-qPCR analysis of the expression of *ThHSFA1* in different transgenic *T. hispida* plants. The expression of *ThHSFA1* was determined under normal control conditions and treatment with 150 mM NaCl or 40 μM ABA for 24 h. The expression level of *ThHSFA1* in VC plants under control conditions was used as the calibrator (designed as 1). VC: the empty pROKII transformed *T. hispida* plants; RNAi: transiently RNAi-silencing *ThHSFA1* in *T. hispida* plants. OE: transiently overexpressing of *ThHSFA1* in *T. hispida* plants. The error bars represent ± SD, which were calculated from multiple biological replicates.

Table S1. Primer sequences used in this study.

Genes	GenBank number	Forward and reverse primers (5'–3')		Reference
**Primers used for RT-qPCR**				
ThWRKY4	JX416193	GCCTTCGTTTATCTTGCCTGCTC	CGATCGGTGCGGGCCTCTTC	Zheng et al., 2013 [7]
ThHSFA1	MW881195	AGCAATCAAGCGATAACC	AGGAGGAATGCCGTCAAG	
Thtubulin		CACCCACCGTTGTTCCAG	ACCGTCGTCATCTTCACC	Zheng et al., 2013 [7]
PagActin		ACCCTCCAATCCAGACACTG	TTGCTGACCGTATGAGCAAG	
**Primers used for ChIP-PCR**				
Pro$_{ThWRKY4}$		TAGATATCTATACTTTAATAC	TGTTTTATGTTACGAATCTG	
Pro$_{Thtubulin}$		TACCATACCCGTATCCGTAC	ACCATACCCGTATCCATATC	

Constructs	Forward and reverse primers (5'–3')	
**Specific primers used for genetic transformation of poplar**		
pMD^{TM}19-T-ThHSFA1	ATGAGTAGCGTTCAAGAAG	TCACATCCTTGTGTTCTCAGC
pDONR207-ThHSFA1	GGGGACAACTTT-GTACAAAAAAGTTGGAATGAG-TAGCGTTCAAGAAG	GGCGGCCGCACAACTTT-GTACAAGAAAGTTGGG-TATCACATCCTTGTGTTCTCAGC
pROKII-ThHSFA1	CTCTAGAGGATCCCCGG-GATGAGTAGCGTTCAAGAAG	TCGAGCTCGG-TACCCGGGTCACATCCTT-GTGTTCTCAGC
pFGC5941-ThHSFA1	ThHSFA1-Cis(AscI)-F: TTGGCGCGCCCCAC-CGGCCTCCATGAAACC	ThHSFA1-Cis(SwaI)-R: CATGATTTAAATCTCACCCCCAG-CAGAATCAG
	ThHSFA1-Anti(PacI)-F: CCTTAATTAACCAC-CGGCCTCCATGAAACC	ThHSFA1-Anti(XbaI)-R: TGCTCTAGACTCACCCCCAG-CAGAATCAG
Genomic DNA PCR	GACGCACAATCCCACTATCC	TCACATCCTTGTGTTCTCAGC
**Primers used for yeast one-hybrid assay**		
pGADT7-Rec2-ThHSFA1	TGGCCATTATGGCCCGG-GATGAGTAGCGTTCAAGAAG	GACATGTTTTTTCCCGGGTCACAT CCTTGTGTTCTCAGC
pHIS2-3×HSE	AATTCCACGAAAAATTCAA-GCACGAAAAATTCAAGCAC-GAAAAATTCAAGGAGCT	CCTTGAATTTTTCGTGCTT-GAATTTTTCGTGCTT-GAATTTTTCGTGG
pHIS2-Pro$_{ThWRKY4}$	ACTATAGGGCGAATTCTAGA-TATCTATACTTTAATAC	GCGAAC-GCGTGAGCTCTGTTTTATGTTAC-GAATCTG
**Primers used for dual-luciferase reporter assay**		
pGreenII 62-SK-ThHSFA1	TCTAGAACTAGTG-GATCCATGAGTAGCGTTCAA-GAAG	GCGTACCGAATTGGTAC-CTCACATCCTTGTGTTCTCAGC
pGreenII 0800-Luc-Pro$_{ThWRKY4}$	GACGGTATCGATAAGCTTTA-GATATCTATACTTTAATAC	TCTAGAACTAGTG-GATCCTGTTTTATGTTACGAATCTG
**Primers used for subcellular localization and transcriptional activation assay**		
pBI121-ThHSFA1-GFP	TCTAGACTGGTACCCGG-GATGAGTAGCGTTCAAGAAG	CTAGTCAGTCGACCCGGG-*TA*CATCCTTGTGTTCTCAGC
pGBKT7-ThHSFA1	CATGGAGGCCGAATTCATGAG-TAGCGTTCAAGAAG	GCAGGTCGAC-GGATCCTCACATCCTT-GTGTTCTCAGC
pGBKT7-ThHSFA1-N	CATGGAGGCCGAATTCATGAG-TAGCGTTCAAGAAG	GCAGGTCGAC-GGATCCAATCTGTCCATCTGATA-TAG
pGBKT7-ThHSFA1-C	CATGGAGGCCGAATTCGTGAAGT ATCAGCCCTTAAT	GCAGGTCGAC-GGATCCTCACATCCTT-GTGTTCTCAGC
